# Plasmon coupling nanorice trimer for ultrahigh enhancement of hyper-Raman scattering

**DOI:** 10.1038/s41598-020-78814-0

**Published:** 2021-01-13

**Authors:** Shuangmei Zhu, Chunzhen Fan, Erjun Liang, Pei Ding, Xiguang Dong, Haoshan Hao, Hongwei Hou, Yuanda Wu

**Affiliations:** 1 Henan Key Laboratory of Electronic Ceramic Materials and Application and College of Science, Henan University of Engineering, Zhengzhou, 451191 China; 2 College of Chemistry, Zhengzhou University, Zhengzhou, 450001 China; 3 School of Physics and Microelectronics and MOE Key Laboratory of Materials Physics, Zhengzhou University, Zhengzhou, 450001 China; 4 Henan Shijia Photons Technology Co., Ltd., Hebi, 458030 China; 5 School of Materials Science and Engineering, Zhengzhou University of Aeronautics, Zhengzhou, 450046 China

**Keywords:** Nonlinear optics, Raman spectroscopy, Nanophotonics and plasmonics

## Abstract

A new tactic that using Ag nanorice trimer as surface-enhanced hyper Raman scattering substrate is proposed for realizing maximum signal enhancement. In this paper, we numerically simulate and theoretically analyze the optical properties of the nanorice trimer consisting of two short nanorices and a long nanorice. The Ag nanorice trimer can excite Fano resonance at optical frequencies based on the strong interaction between the bright and the dark mode. The bright mode is attributed to the first longitudinal resonance of the short nanorice pair, while the dark mode originates from the third longitudinal mode resonance of the long nanorice. The electric field distributions demonstrate that the two resonances with the largest field strength correspond to the first-order resonance of the long nanorice and the Fano resonance of the trimer, respectively. Two plasmon resonances with maximum electromagnetic field enhancements and same spatial hot spot regions can match spectrally with the pump and second-order Stokes beams of hyper Raman scattering, respectively, through reasonable design of the trimer structure parameters. The estimated enhancement factor of surface-enhanced hyper Raman scattering can achieve as high as 5.32 × 10^13^.

## Introduction

Hyper-Raman scattering (HRS) is an inelastic sum-frequency scattering from two photons, which is different from normal Raman scattering (NRS) based on a single photon scattering. The two photons of frequency ν are inelastically scattered from a ground state to a virtual state with energy equal to 2ν ± ν_vib_ (corresponding to Stokes and anti-Stokes scattering)^1,2^. Since it was discovered, HRS effect has aroused great interests due to its special selection rule^3^. However, HRS is a two-photon process and has a tiny scattering cross-section of about 10^–6^ of NRS, so the experimental testing was very difficult^4^. It was not until the discovery of surface-enhanced Raman scattering (SERS) phenomenon that HRS was considered as an applied spectroscopy technique. Surface enhanced hyper Raman scattering (SEHRS) proposed after the discovery of SERS is a two-photon excitation, which is similar to SERS^5^. HRS is considered to be a more sensitive spectroscopic technique to changes in the surface environment than NRS due to its better insight into molecular structure and interactions^6–9^. SEHRS can display some information that is not shown in SERS data, for instance the mode-specific enhancements from resonance with different electronic states^10,11^, the orientation of molecules on metal surfaces^12^, trace analysis of nonresonant molecules^13^ and the local chemical effects^14^. With the evolution of simulation experiment and theoretical research, SEHRS has attracted a lot of attention and has good application development in many fields^15–20^.

A very peculiar outcome of electromagnetic (EM) coupling is the Fano-type resonance (FR) in complex metallic plasmonic nanostructures, which generally has obvious asymmetric linear properties. FR originates from destructive interference between the superradiant (bright) and the subradiant (dark) mode in complex metallic plasmonic nanostructures. Because of the significant applications, it has attracted extensive attention in recent years, such as slow-light optical device, optical switching, SERS, biological sensors, nonlinear, electromagnetically induced transparency and so on^21–28^. Appropriate engineering studies on plasmon resonance in complex photonic structures can provide effective strategies for enhancing local electromagnetic field, customizing nanoscale spectral response, and optimizing Raman response of the material. One effective solution is to generate FR in a coupled plasmonic system. Recently, FRs have been demonstrated in the dolmen-type slab structures^29,30^ and the nonconcentric ring/disk cavity^31^, respectively. Plasmonic nanostructure assemblies consisting of three^32^, four^33^ and even more nanoparticle aggregates^34^ also exhibit very strong FR, when each constituted nanoparticle has sufficiently small interparticle separation.

Recently, we have designed and researched a kind of SEHRS substrate in theory, which was consisted of Ag nanorice^35^. To achieve a significant amplification of the HRS signal, the two plasmon resonance modes in this nanostructure were matched with the excitation and the second-order Stokes lights, respectively. The mainly factor to achieve this purpose is that the Ag nanorice can excite multiple plasmon resonances with strong local electromagnetic effect at same hotspot positions. At the same time, the plasmon resonance frequencies match with the excitation or the second-order Stokes lights.

In this article, based on our previous research, we studied the extinction performance of Ag nanorice trimers and provided a new strategy for the layout of plasmonic SEHRS substrate. By changing the structural parameters, the resonance spectra have been flexibly adjusted, and a plasmonic substrate for SEHRS has been designed and optimized. The nanorice trimer structure exhibits an extinction spectrum with Fano-type profile. The influences of different structural parameters on the enhancement of HRS spectroscopy have been discussed. Two excited plasmon resonance modes with large field enhancements corresponding to the first-order resonance of the long nanorice and the FR of the trimer spectrally match with the excited light and the second-order Stokes light, respectively, at the same hot spots. The calculated maximum enhancement factor (EF) of SEHRS substrate can be up to 5.32 × 10^13^, and is almost four orders of magnitude higher than that of our study on Ag nanorice substrate. We expect this work is not only to provide promising applications for single molecule detection, but also to solve the problem for SEHRS enhancement mechanism and related field development.

## System description

**Figure 1:**
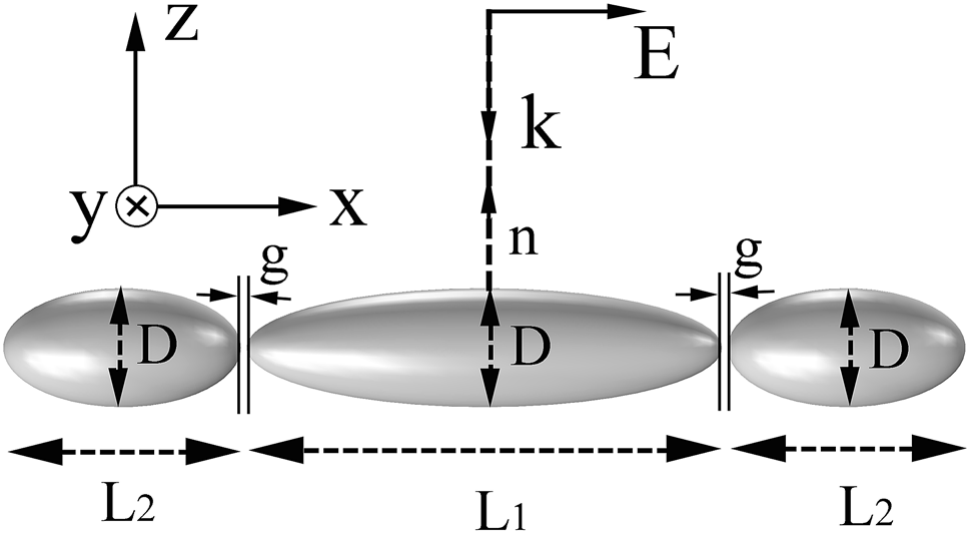
Excitation model of the silver nanorice trimer. The trimer locates in the x–y plane, with its normal direction n along the negative direction of the z axis. Linearly polarized light illuminates this trimer with normal incidence.

Numerical simulations were carried out by using COMSOL Multiphysics based on the finite element method (FEM). Perfect matched layer is hired at the surrounding boundaries of the silver nanorice trimer to avoid spurious reflections. The relative permittivity of silver nanorice was obtained from the data of Johnson and Christy^36^. Extinction, scattering, absorption cross-sections were computed on the basis of the formulations in Ref.^37^. In order to simplify the calculation, the surrounding dielectric environment was supposed to be air with the refractive index of *n* = 1. In a real experimental situation, the introduction of dielectric substrates will make the resonance wavelength longer and the line width slightly wider, but it does not modify the optical properties of the trimer^38,39^. The cross-sections and the electric field distributions of the silver nanorice trimer were calculated, whose structure is shown in Fig. 1. The three nanorices are ellipsoids with length *L*_2_, *L*_1_, and *L*_2_, and diameter *D*, respectively. The distances between the long nanorice and the two short nanorices are *g*. A plane electromagnetic wave is incident along the negative *z*-direction with the electric field polarized along the *x*-direction.

## Results and discussions

### Spectral tunabilty of FR

**Figure 2:**
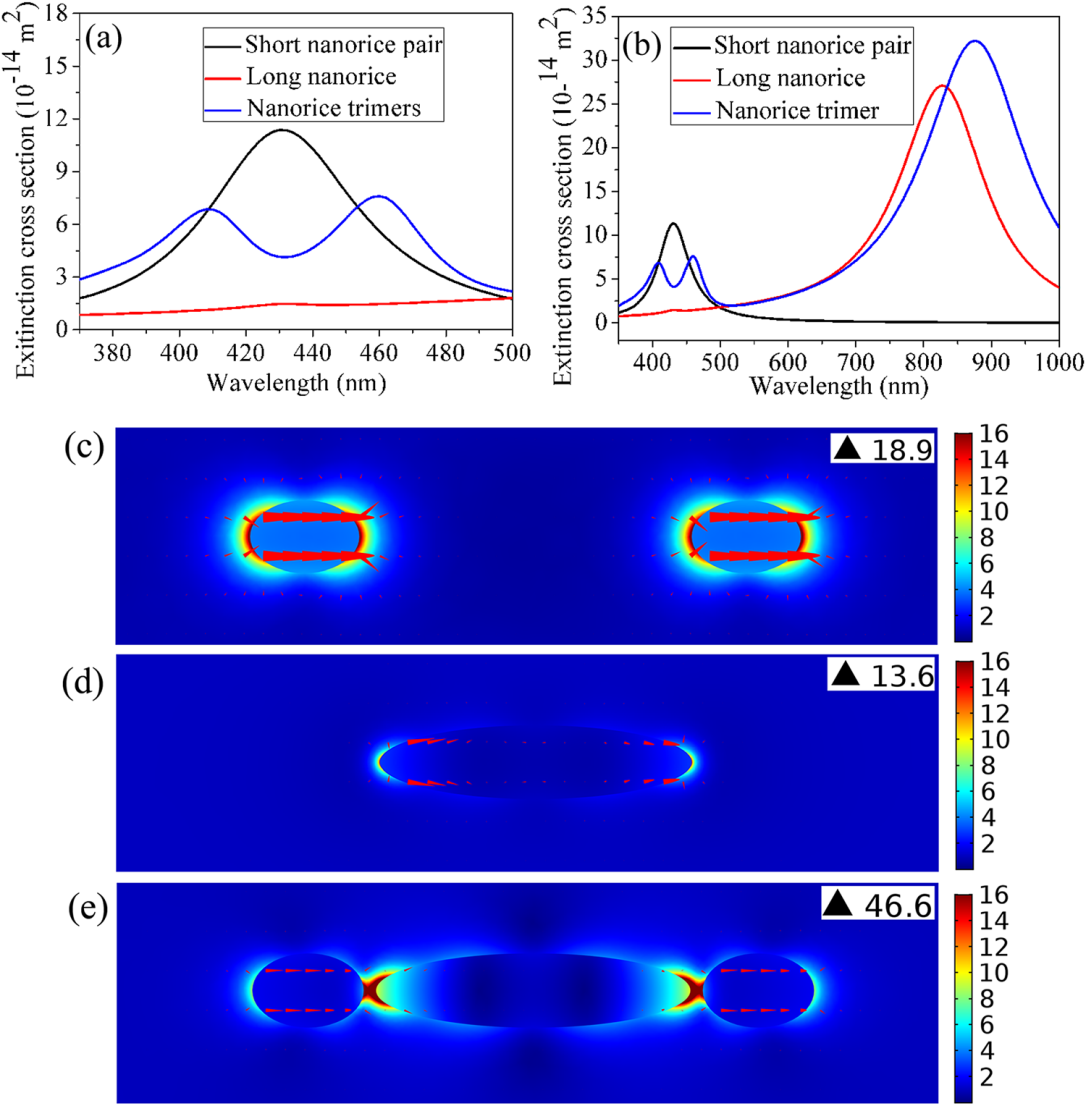
(**a**) Extinction cross-sections in the wavelength range from 370 to 500 nm of a silver nanorice trimer (L_1_ = 254 nm, D = 60 nm, L_2_ = 90 nm, g = 10 nm), short nanorice pair (L = L_2_ = 90 nm, D = 60 nm, g = 274 nm) and long nanorice (L = L_1_ = 254 nm, D = 60 nm). (**b**) Extinction cross-sections in the wavelength range from 350 to 1000 nm of a silver nanorice trimer, short nanorice pair and long nanorice. The field distributions of the short nanorice dimer, long nanorice and silver nanorice trimer at wavelengths of 430 nm are displayed in (**c**–**e**). The arrows and the size of the arrows indicate the direction and the intensity of charge oscillation, respectively. Scale factor of the arrows is 6 × 10^–5^.

The localized electromagnetic field enhancement arised from plasmon resonance contributes greatly to the enhancement of HRS, especially when the probed molecules are the locations of the hot spots. The extinction cross-section (black curve) of one individual short Ag nanorice pair (D = 60 nm, L_2_ = 90 nm and the gap = 274 nm), as shown in Fig. 2a. The nanorice pair can be thought of as two independent nanoparticles, because the spacing between them is large enough. The strong resonance at about 430 nm is a dipolar resonance of short nanorice pair which can regard as a bright mode. For one individual long Ag nanorice (D = 60 nm and L_2_ = 254 nm), the very weak resonance occurring at about 430 nm (red curve) is a hexapole plasmon resonance (Supplementary Fig. S1), which can regard as a dark mode in Fig. 2a. Figure 2a also shows the extinction cross-section (blue curve) of the nanorice trimer formed a nanorice pair bright mode and a nanorice dark mode. The short and long nanorices are the same with the nanorice trimer, and the distances between the long nanorice and the short nanorice are g = 10 nm. As compared with the extinction cross-section spectrum of the short nanorice pair, it can be found that the spectrum of the trimer is greatly ameliorated, which confirms that the dark mode of the long nanorice is excited by the bright mode of the short nanorice pair. It is discovered that a dip emerges in the extinction cross-section spectrum of the nanorice trimer, which is caused by Fano interference in the nanorice timer system. According to reference^40^, when the light frequencies are resonant with both bright and dark modes, the bright mode of the short nanorice pair will be aroused by two different paths: ǀI> → ǀB> and ǀI> → ǀB> → ǀD> → ǀB>, where ǀI>, ǀB>, and ǀD˃ are excitation light, bright mode, and dark mode, respectively. Destructive interference occurs between the two pathways when the incident light frequency is approaching the resonant frequency, which leads to the polarization cancellation of the bright mode and a corresponding Fano dip in the extinction spectrum. It is also found that near 458 nm or 408 nm, the small net dipole moment of the long nanorice will hybridize with the dipole mode of the short nanorice pair. Eventually, the bandwidth of trimer extinction spectrum is wider than that of the individual short nanorice pair. When the wavelength range is from 350 to 1000 nm, the extinction cross-sections of individual short Ag nanorice pair, individual long Ag nanorice, and the nanorice trimer, respectively, are shown in Fig. 2b. The extinction spectrum of the silver nanorce trimer show that the dipole peak shifts toward long wavelengths under the influence of nanorice pair as compared with that of single long nanorice. The electric field distributions of one a bright mode nanorice pair, one dark mode nanorice, and the nanorice trimer at wavelength of 430 nm are shown in Fig. 2c–e respectively. As can be seen from Fig. 2c, the “hot spots” are located at the end of each nanorices along the long axis and the maximum value of the electric field is 18.9 at 430 nm. From Fig. 2e shows that the enhancement of electromagnetic field intensity of the bright mode nanorice pair at the Fano dip is greatly suppressed at wavelength of 430 nm, which was set down to the polarization cancellation in the Fano resonance. The solid curves display the extinction, absorption, and scattering spectra of the silver nanorice trimer and the dashed curves reveal that of short nanorice pair in Supplementary Fig. S2. It can be seen that the dipole resonances of the three cross-sections of short nanorice pair are in the same wavelength range but the Fano-dip positions of the three cross-sections are in the different wavelength positions. The Fano-dip position of the extinction cross-section show better fidelity to the dipole resonance position of the nanorice pair, compared with the absorption and scattering cross-sections of the silver nanorice trimer, and a clear dip at around 430 nm is seen.

**Figure 3:**
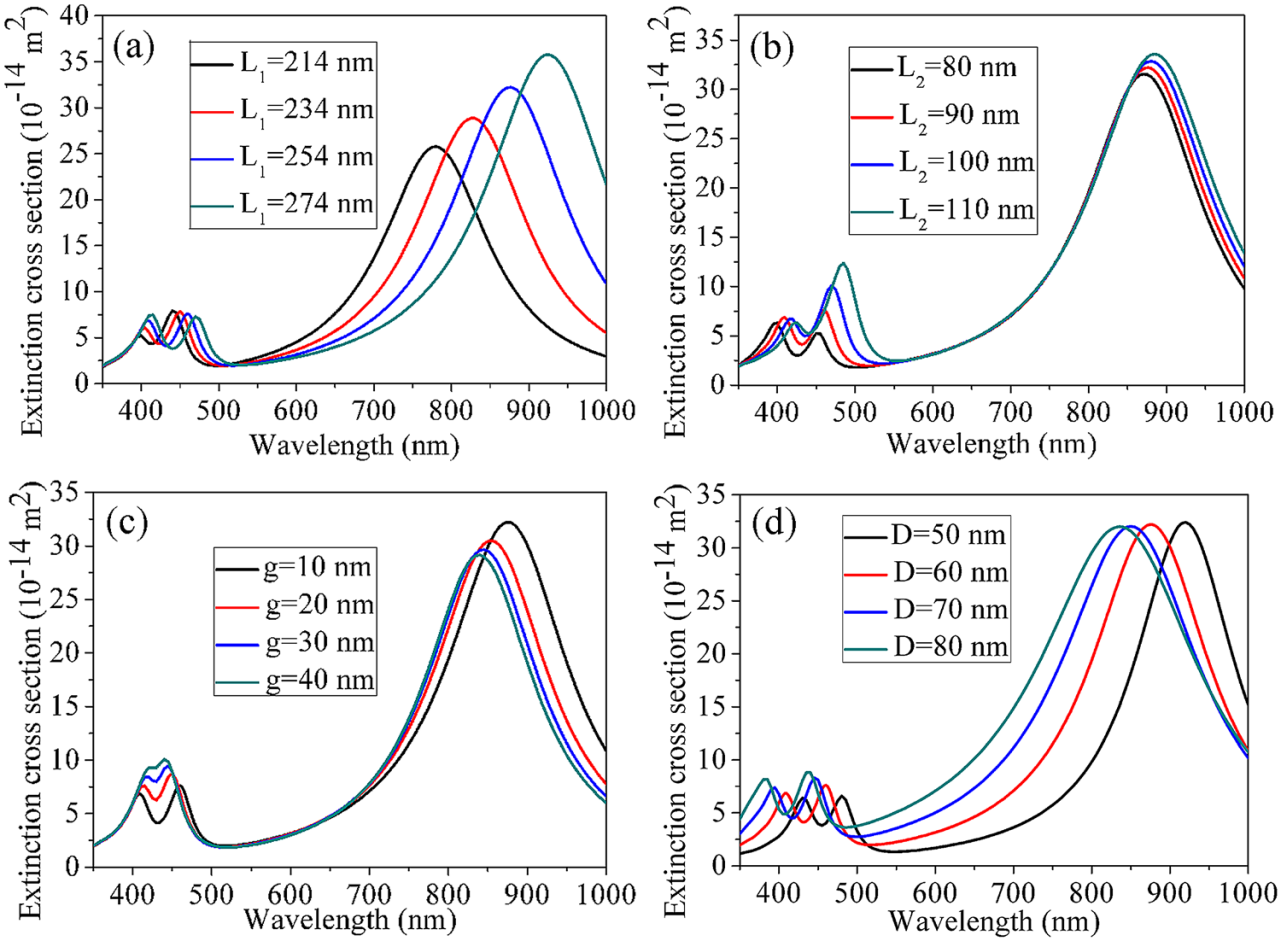
Extinction cross-sections of the trimers with different configuration parameters. (**a**) Varying the length of a long nanorice (*L*_1_) with fixed *g* = 10 nm, *L*_2_ = 90 nm, *D* = 60 nm. (**b**) Varying the length of a short nanorice pair (*L*_2_) with fixed *g* = 10 nm, *L*_1_ = 254 nm, *D* = 60 nm. (**c**) Varying the gap (*g*) with fixed *L*_1_ = 254 nm, *L*_2_ = 90 nm. (**d**) Varying the diameter of the nanorice trimer (*D*) with fixed *g* = 10 nm, *L*_1_ = 254 nm, *L*_2_ = 90 nm.

The spectral position and the plasmon coupling strength can be effectively optimized by adjusting the geometrical parameters of silver nanorice trimer, including the Length (L_1_) of long nanorice, the length (L_2_) of short nanorice pair, the gap (g) of nanorices and the diameter (D). Figure 3 displays the extinction cross-section of the silver nanorice trimer with different geometrical parameters. When the length of the dark mode nanorice is redshifts from 214 to 274 nm by increasing the long nanorice length and the short nanorices pair length at 90 nm are fixed, the peak intensity on the right side of the Fano dip decreases slightly while the one on the left side increases (see Fig. 3a). The Fano dip and the two peak intensity of the extinction cross-section shift toward long wavelengths, with the redshift more obvious for the dipole resonance coupling mode of long nanorice and short nanorice dimer. In Fig. 3b, as the bright mode wavelength redshifts by adding the lengths of the vertical axis of the two short nanorices from 80 to 110 nm and fixing the length of long nanorice at 254 nm, the peak intensities of extinction cross-section on both sides of the Fano dip show opposite behaviors. The Fano dip and the left peak of the Fano resonance and dipole resonance coupling mode of long nanorice and short nanorice dimer shift toward long wavelength, but the redshift is more pronounced for the right peak of the Fano resonance. The plasmon coupling strength around Fano interferences can be regulated by changing the gaps between silver nanorices (see Fig. 3c). The coupling strength increases when the gaps between silver nanorices decrease. Then, the dip of Fano resonance becomes wider and deeper, according with Ref.^41^. In addition, compared with the extinction spectrum of the short nanorice dimer, the one of the trimer is wider. These phenomena are caused by the presence of a wide spectrum rather than a single frequency near the dark mode resonance involved in Fano interference and plasmon hybridization. In addition to the coupling strength of Fano resonance, the width and depth of spectral lines are also affected by the gaps of the nanorice trimer. In Fig. 3d, when the diameter of the nanorice trimer is increased from 50 to 80 nm and the long and short nanorices length are fixed at 254 nm and 90 nm, the bright mode and dark mode wavelength blueshift and the extinction cross-section peak intensities on both sides of the Fano dip remain unchanged basically. The Fano dip and dipole resonance coupling mode of long nanorice is blue shifted, but the blueshift is more remarkable for dipole resonance coupling mode of long nanorice and short nanorice dimer.

### SEHRS enhancement

In recent years, the development of active plasmonic substrates has provided huge boost for the study of surface-enhanced spectroscopy. HRS has also been extensively studied in the single molecule detection^20^ and the design of active substrates^35^. We know that it is a second-order nonlinear optical detection^42^. When the incident light with frequency ν interacts with the medium, the medium absorbed two photons with frequency ν, and also emitted one photon with frequency *ν*_s_ (*ν*_s=_2*ν* ± *ν*_vib_), where *ν*_vib_ was the molecular vibration frequency. At the resonance position, the HRS signal will be resonant enhanced. The enhanced HRS signal can be achieved with a metal plasmonic substrate, which can be described as SEHRS. The EF of electric field is expressed as^43^:

1 $$\mathop {EF}\nolimits_{SEHRS}^{EM} = \left| {{{E(\nu )} \mathord{\left/ {\vphantom {{E(\nu )} {E_{0} (\nu )}}} \right. \kern-\nulldelimiterspace} {E_{0} (\nu )}}} \right|^{4} \left| {{{E(\nu_{s} )} \mathord{\left/ {\vphantom {{E(\nu_{s} )} {E_{0} (\nu_{s} )}}} \right. \kern-\nulldelimiterspace} {E_{0} (\nu_{s} )}}} \right|^{2} = \left| {g(\nu )} \right|^{4} \left| {g(\nu_{s} )} \right|^{2}$$

The *g*(*ν*) and *g*(*ν*_*s*_) are the enhancements of the localized electric fields at the frequencies of incident (|*E*(ν)/*E*_0_(ν)|) and scattering (|*E*(ν_*s*_)/*E*_0_(ν_*s*_)|) resonance, respectively. This implies that the HRS signal enhancement of local fields is determined by two characteristic frequencies. An optimal SEHRS substrate requires (1) high coinstantaneous electric-field enhancement for the incident light, second-order Stokes photons at the corresponding frequencies and (2) the enhanced electric-fields localized at the same spatial regions at two different frequencies, commonly known as “hot spots”, being, from Eq. (1). Whereas, the frequency of the incident and scattered light often can only enhance one of them in the regular SEHRS substrate, that is, electromagnetic enhancement contributes are either |*g*(*ν*)|^4^ or |*g*(*ν*_*s*_)|^2^. Therefore, for the regular SEHRS substrate^44,45^, the two conditions are not easy to implement at the same time. Thus, a suitable plasmonic substrate will provide strong electromagnetic field relying on two characteristic frequencies, which realizes the HRS signal enhancement. If a metal nanostructure has two different resonant modes and occurs at the same spatial location, it may be a viable SEHRS substrate to achieve a strong field enhancement. An optimal SEHRS substrate also requires high coinstantaneous electric-field enhancement for the incident light, second-order Stokes photons at the corresponding frequencies. Simultaneously, surface plasmon resonance plays an important role in the excitation of large electromagnetic fields near metal surfaces and the field localization is usually caused by structural coherence^46^. So, a plasmonic Fano resonance SEHRS substrate with high coinstantaneous electric-field enhancement for the incident light, second-order Stokes photons at the corresponding frequencies and the enhanced electric-fields localized at the same spatial regions is better than other systems.

**Figure 4:**
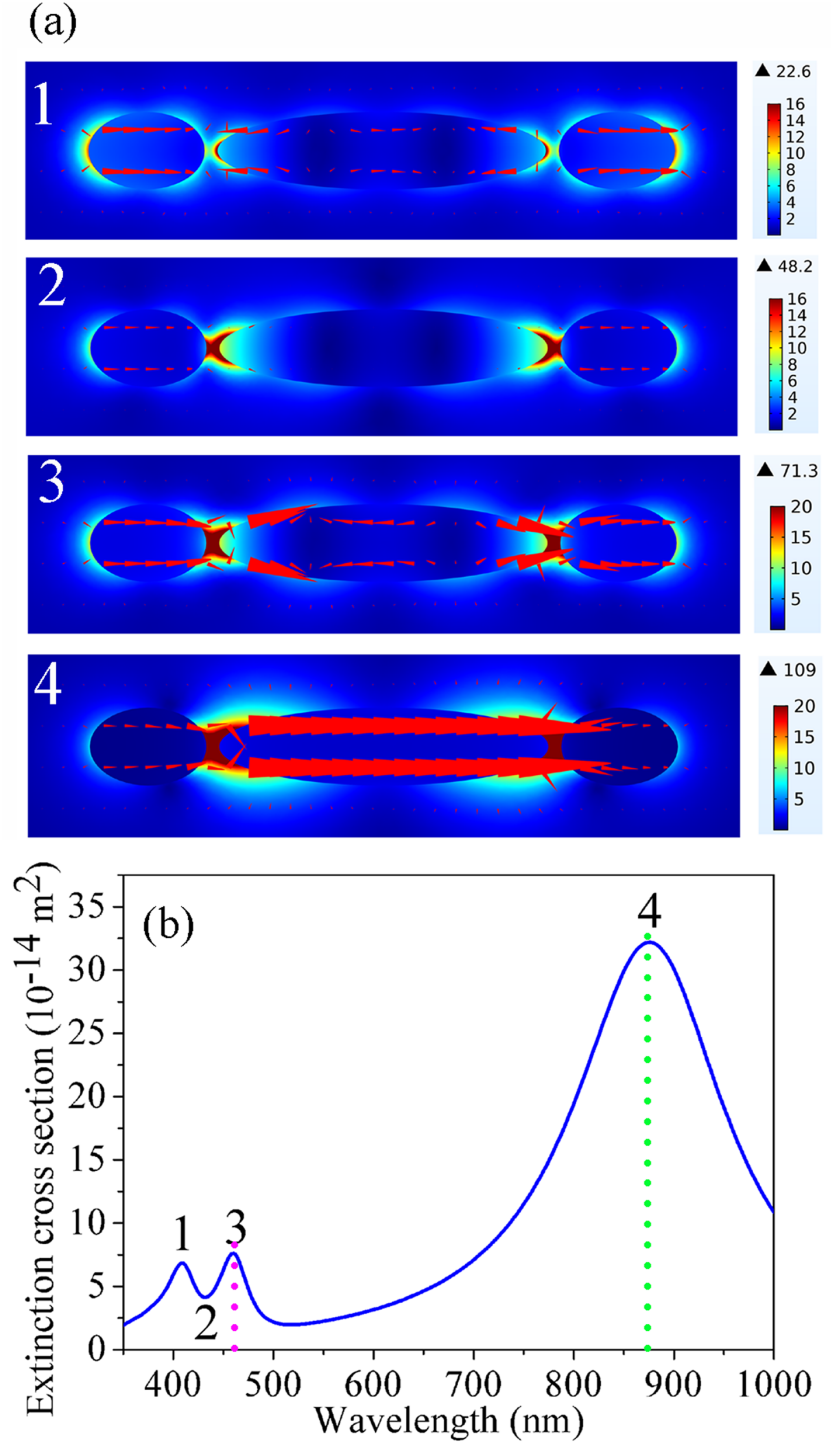
(**a**) Electric field and current flow distributions of the silver nanorice trimer at the positions 1, 2, 3 and 4 in (**b**), respectively. (**b**) Extinction spectrum of the silver nanorice trimer (L_1_ = 254 nm, L_2_ = 90 nm, D = 60 nm, g = 10 nm) for enhancing the 997 cm^−1^ mode with 876 nm excitation light. Here, the mode 4 and mode 3 resonance are matched to the excitation light (green dotted line, 876 nm) and second-order Stokes (pink dotted line, 458 nm) wave, respectively.

As depicted by Fig. 4, the distributions of electric field and current flow and the extinction cross-section of a nanorice trimer (L_1_ = 254 nm, D = 60 nm, L_2_ = 90 nm, g = 10 nm) formed a super-radiation mode nanorice pair and a sub-radiation mode nanorice. The electric field distributions of the trimer at wavelengths of 408, 430, 458, and 876 nm, corresponding to the positions 1, 2, 3 and 4 in Fig. 4b, are shown in Fig. 4a-1, a-2, a-3, and a-4, respectively. Figure 4b highlights the extinction spectrum of a nano-trimer formed a bright mode nanorice pair and a dark mode nanorice. Near the Fano dip, the two electron oscillations of the long nanorice and the nanorice pair are opposite and their strengths are basically the same, and the collective electron oscillation of the nanorice pair is neutralized because of Fano interference (see Fig. 4a-2). Therefore, the whole nano-trimer is dark because there has no net dipole moment on it. There is a strong dipole plasmon mode on the short nanorice pair at 458 nm (see Fig. 4a-3). Because the electron oscillations in the middle of the long nanorice are quite counterbalanced by those at the ends, the long nanorice is still an approximate dark mode although there is a small net dipole moment on it. Therefore, the whole nanorice trimer is bright. Similar to what happened at 458 nm, the trimer is bright at 408 nm (see Fig. 4a-1). There is a super-strong dipole resonance coupling mode of long nanorice and short nanorice dimer at 876 nm (see Fig. 4a-4). From the electric field distribution (Fig. 4a), it can be found that the field intensity is the strongest at 458 nm and 876 nm in the gap position.

To evaluate HRS enhancement performance of the Ag nanorice trimer, so now we calculate the SEHRS electromagnetic EF for a Raman mode of meta-hydroxybenzoic acid molecules at 997 cm^−1^. Though the optimization of the geometrical parameters of the model, we tune the position of the dipole resonance coupling mode just corresponding to the wavelength of exciting light, and the right side of the Fano dip is consistent with wavelength of the second-order stokes scattering wave (see Fig. 4b). The calculated EF of SEHRS is 7.18 × 10^11^ (EF_SEHRS_ = 109^4^ × 71.3^2^ ≈ 7.18 × 10^11^).

In our design of the silver nanorice trimer structure, when *L*_1_ and *L*_2_ are adjusted to be unequal, two different plasmon resonances can be achieved. There is a need to localize of the two resonant frequencies in the same “hotspot” position, in order to realize large HRS enhancement. Based on the study of optical features of nanorice trimer structures, when the exciting light is assumed in the near-infrared wavelength range, we have acquired HRS enhancement in the “fingerprint” spectral region (200–2000 cm^−1^) by modulating the *L*_1_, *L*_2_, *g* and *D*, as shown in Table 1. The SEHRS EFs can reach to order of 10^10^–10^12^ based on Fano resonance. The EF decreases when *D* and *g* increase, but the EF has the opposite change trend when *L*_1_ and *L*_2_ increase. These results suggest that the silver nanorice trimer structure have great promise in single molecule detection.

**Table 1:** Evaluation of the SEHRS EF with the different configuration parameters.

Structure	L_1_ (nm)	L_2_ (nm)	g (nm)	D (nm)	Excitation		Second-order stokes		EF_SEHRS_	Raman shift (cm^-1^)
					λ_ex_ (nm)	|*g*(*ν*)|^4^	λ_s_ (nm)	|*g*(*ν*_*s*_)|^2^		
1	234	90	10	60	834	101^4^	448	65^2^	4.40 × 10^11^	1659
2	254	100	10	60	884	112^4^	468	72.6^2^	8.29 × 10^11^	1257
3	254	110	10	60	890	114^4^	482	68.3^2^	7.88 × 10^11^	1725
4	254	90	10	60	876	109^4^	458	71.3^2^	7.18 × 10^11^	997
5	254	90	20	60	860	80.8^4^	446	51.7^2^	1.14 × 10^11^	834
6	254	90	30	60	844	72.4^4^	440	43.8^2^	5.27 × 10^10^	969
7	254	90	10	50	920	157^4^	480	91.9^2^	5.13 × 10^12^	906
8	274	90	10	60	924	119^4^	470	76.0^2^	1.16 × 10^12^	368
9	254	90	10	70	854	83.3^4^	444	58.1^2^	1.62 × 10^11^	897

Here, the g(v) and g(v_s_) are the enhancements of the localized electric fields (|E/E_0_|) at the excitation and second-order Stokes wavelengths, respectively. The monitored position of the electric field enhancements is located in the central gap region of the nanorice trimer in the x–y plane. The SEHRS EF is calculated by formula (1).

In fact, the SEHRS enhancements effect can be further optimized by reducing the gap distance of the Ag nanorice trimer. The near field coupling between the constituent nanostructures increases as the gap distance is reduced, so the amplitude of electronic field “hot spots” will increases significantly^47–51^, thereby resulting in a significant increase of SEHRS EF. We further evaluate the SEHRS EF of a trimer with L_1_ = 254 nm, L_2_ = 90 nm, D = 50 nm*,* and g = 5 nm, for the 906 cm^−1^ Raman mode with an excitation wavelength of 942 nm (Supplementary Fig. S3). The result show that the maximum SEHRS EF can reach ~ 5.32 × 10^13^, and obtain significant increase relative to the value occurring in the trimer with the 10 nm gap distance. Ag nanorice trimers with sub 10 nm gap can be fabricated recurring to electron beam lithography nanopatterning technique^52–54^.

## Conclusion

In this article, we have discussed and analyzed a Fano-resonance plasmonic substrate consisting of silver nanorice trimer structure and explored strategies for bringing the electric field “hot spots” of the photons with different frequencies involved in SEHRS process to the same spatial position. The results show that the electric field “hot spots” pointing at a specific spectral position of plasmon resonance can be tuned actively by changing structural parameters of the silver nanorice trimer. The silver meso-flowers monolayer SEHRS substrate exhibits a delightful EF value (5.32 × 10^13^), and can reach the sensitivity of single-molecules detection. The Fano-resonance plasmonic substrate developed in this study could be applied to other nonlinear spectroscopy, stimulated Raman scattering and multiphoton imaging etc.

## Supplementary information

Supplementary Figures.
