# Author Correction: Plasmon coupling nanorice trimer for ultrahigh enhancement of hyper-Raman scattering

**DOI:** 10.1038/s41598-021-88554-4

**Published:** 2021-04-16

**Authors:** Shuangmei Zhu, Chunzhen Fan, Erjun Liang, Pei Ding, Xiguang Dong, Haoshan Hao, Hongwei Hou, Yuanda Wu

**Affiliations:** 1 Henan Key Laboratory of Electronic Ceramic Materials and Application, College of Science, Henan University of Engineering, Zhengzhou, 451191 China; 2 College of Chemistry, Zhengzhou University, Zhengzhou, 450001 China; 3 School of Physics and Microelectronics, MOE Key Laboratory of Materials Physics, Zhengzhou University, Zhengzhou, 450001 China; 4 Henan Shijia Photons Technology Co., Ltd, Hebi, 458030 China; 5 School of Materials Science and Engineering, Zhengzhou University of Aeronautics, Zhengzhou, 450046 China

Correction to: *Scientific Reports* 10.1038/s41598-020-78814-0, published online 13 January 2021

The Acknowledgements section in this Article is incomplete.

"National Science Foundation of China (NSFC) (11604079); Science and Technology Program of Henan Province (192102210198); China Postdoctoral Science Foundation funded project (2018M632793); Foundation for University Young Key Teacher Program by Henan Province, China (2017GGJS155); Doctoral Fund Project of Henan Institute of Engineering (D2017023); the Key Scientific Research Projects of Institutions of Higher Learning in Henan Province (20A140006)."

should read:

"National Science Foundation of China (NSFC) (11604079); Science and Technology Program of Henan Province (192102210198 and 212102310903); China Postdoctoral Science Foundation funded project (2018M632793); Foundation for University Young Key Teacher Program by Henan Province, China (2017GGJS155); Doctoral Fund Project of Henan Institute of Engineering (D2017023); the Key Scientific Research Projects of Institutions of Higher Learning in Henan Province (20A140006)."

