# Supplementary material for: Plasmon coupling nanorice trimer for ultrahigh enhancement of hyper-Raman scattering
Source: Sci Rep. 2021 Jan 13;11:1230. doi: 10.1038/s41598-020-78814-0 (PMC7806829; doi:10.1038/s41598-020-78814-0)
Supplement: Supplementary file 1 — Supplementary Figures. [file 41598_2020_78814_MOESM1_ESM.pdf]

Supplementary Information

Plasmon coupling nanorice trimer for ultrahigh enhancement of hyper-Raman scattering

Shuangmei Mei[1,2,4], Chunzhen Fan[3], Erjun Liang[3,*] , Pei Ding[5,*] , Xiguang Dong[1], Haoshan Hao[1], Hongwei Hou[2], Yuanda Wu [4]

[1] *Henan Key Laboratory of Electronic Ceramic Materials and Application and College of Science, Henan University of Engineering, Zhengzhou 451191, China;*

[2] *The College of Chemistry and Molecular Engineering, Zhengzhou University, Zhengzhou 450001, China*

[3] *School of Physical Science and Engineering and Key Laboratory of Materials Physics of Ministry of Education of China, Zhengzhou University, Zhengzhou 450052, China*

[4] *Henan Shijia Photons Technology Co., Ltd., Hebi 458030, China*

[5] *School of Materials Science and Engineering, Zhengzhou University of Aeronautics, Zhengzhou 450046, China*

Corresponding Author email: ejliang@zzu.edu.cn; peiding76@163.com

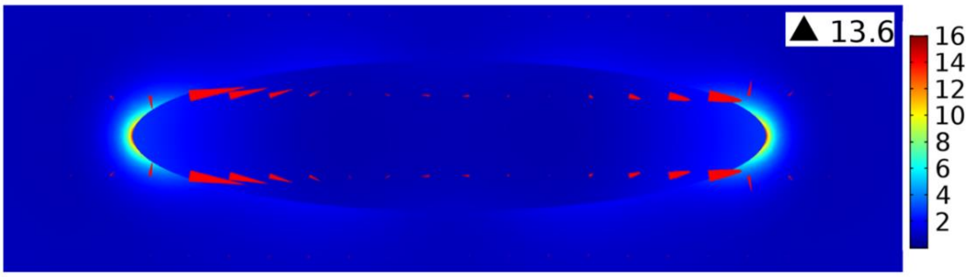

Supplementary Fig. S1. Enlarged view of the field distributions and charge oscillations of the long nanorice (D=60 nm and L_2=254 nm) at wavelengths of 430 nm. The arrows and the size of the arrows indicate the direction and the intensity of charge oscillations, respectively. Scale factor of the arrows is 6×10^{-5}.

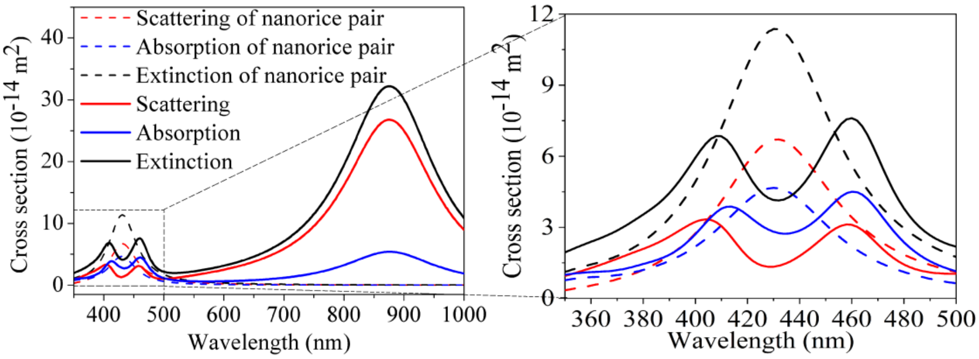

Supplementary Fig. S2. Cross-sections of a silver nanorice trimer (L_1=254 nm, D=60 nm, L_2=90 nm, g=10 nm) in the wavelength range from 350 to 1000 nm and cross-sections of the trimer in the wavelength range from 350 to 500 nm. The dotted lines represent the extinction, absorption, and scattering spectra of short nanorice pair.

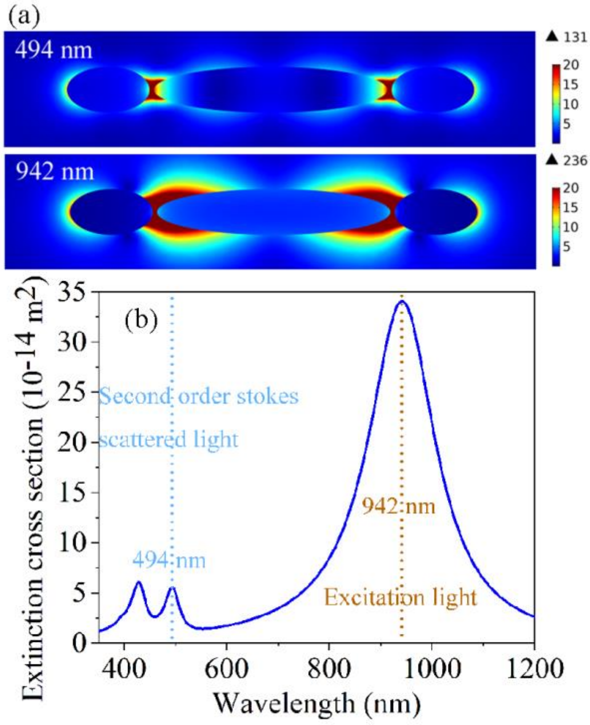

Supplementary Fig. S3. (a) Electric field distributions of the silver nanorice trimer (L_1=254 nm, D=50 nm, L_2=90 nm, g=5 nm). (b) Extinction spectrum of the silver nanorice trimer for enhancing the 906 cm^{-1} mode with 942 nm excitation light. Here, the resonance modes at 942 nm and 494 nm are matched to the excitation light and second-order Stokes wave, respectively.
